# Succinate Dehydrogenase, Succinate, and Superoxides: A Genetic, Epigenetic, Metabolic, Environmental Explosive Crossroad

**DOI:** 10.3390/biomedicines10081788

**Published:** 2022-07-25

**Authors:** Paule Bénit, Judith Goncalves, Riyad El Khoury, Malgorzata Rak, Judith Favier, Anne-Paule Gimenez-Roqueplo, Pierre Rustin

**Affiliations:** 1 NeuroDiderot, Inserm, Université Paris Cité, F-75019 Paris, France; paule.benit@inserm.fr (P.B.); malgorzata.rak@inserm.fr (M.R.); 2 Paris Centre de Recherche Cardiovasculaire (PARCC), Inserm, Université Paris Cité, F-75015 Paris, France; judith.goncalves@inserm.fr (J.G.); judith.favier@inserm.fr (J.F.); 3 Department of Pathology and Laboratory Medicine, Neuromuscular Diagnostic Laboratory, American University of Beirut Medical Center, Beirut 1107 2020, Lebanon; re70@aub.edu.lb; 4 Département de Médecine Génomique des Tumeurs et des Cancers, Assistance Publique-Hôpitaux de Paris (AP-HP), Hôpital Européen Georges Pompidou, F-75015 Paris, France; anne-paule.gimenez-roqueplo@aphp.fr

**Keywords:** mitochondria, respiratory chain, Krebs cycle, succinate, cancer, encephalopathy, SDH, SDHI, mitotoxic, oxytoxic, pesticides

## Abstract

Research focused on succinate dehydrogenase (SDH) and its substrate, succinate, culminated in the 1950s accompanying the rapid development of research dedicated to bioenergetics and intermediary metabolism. This allowed researchers to uncover the implication of SDH in both the mitochondrial respiratory chain and the Krebs cycle. Nowadays, this theme is experiencing a real revival following the discovery of the role of SDH and succinate in a subset of tumors and cancers in humans. The aim of this review is to enlighten the many questions yet unanswered, ranging from fundamental to clinically oriented aspects, up to the danger of the current use of SDH as a target for a subclass of pesticides.

## 1. Introduction

As well as other Krebs cycle enzymopathies, succinate dehydrogenase (SDH) deficiency has long been held to be incompatible with human life [1]. Yet, thanks to genetic investigations grounded in clinical features and biochemical evidence of enzyme deficiencies, one year after fumarase deficiency in 1994 [2], SDH deficiency was definitively recognized as a pathology, although considered to be extremely rare [3]. Nowadays in 2022, SDH deficiency has been finally identified in a whole spectrum of human diseases resulting in unexpected and distant phenotypes [4]. In return, this shed light on the unanticipated roles of SDH and its substrate, succinate, in human physiology and more generally in living organisms. Over the years, a number of reviews have covered most aspects of the physiological and pathological features associated with this enzyme and the associated metabolic segments in a large collection of living organisms [5,6,7,8,9,10,11,12,13,14,15,16,17,18,19,20,21,22,23,24,25,26,27,28,29,30,31,32,33,34,35,36].

Prompted by recent developments in somewhat distant scientific fields, we thought it useful to gather and analyze accessible data to highlight questions that remain open. Thus, we cover in this review the biochemical, metabolic, genetic, epigenetic, and clinical aspects related to SDH and succinate, up to the danger that represents the use of SDH-poisoning substances which, at variable concentrations and alongside other mitotoxic (targeting mitochondrial functions) and oxytoxic (targeting antioxidant defenses and/or increasing oxidative stress) pesticides, impregnate nature and the organisms living therein.

## 2. The Succinate Crossroad: Enzymes and Metabolites Stakeholders

Thanks to the work of a few outstanding biochemists in the 1950s, the main recognized functions of SDH (EC 1.3.5.1) in the mitochondria of aerobic organisms were unraveled. SDH was found to play a role in both the Krebs cycle and the respiratory chain (RC) [32,37,38,39]. In animals, as in most living organisms, SDH has no cellular isoform, unlike most enzymes of the Krebs cycle, except citrate synthase and succinyl CoA ligase. Hence, in the case of dysfunction or deficiency, this reduces the possibility for metabolic bypasses.

### 2.1. The Succinate Dehydrogenase Enzyme

**Figure 1:**
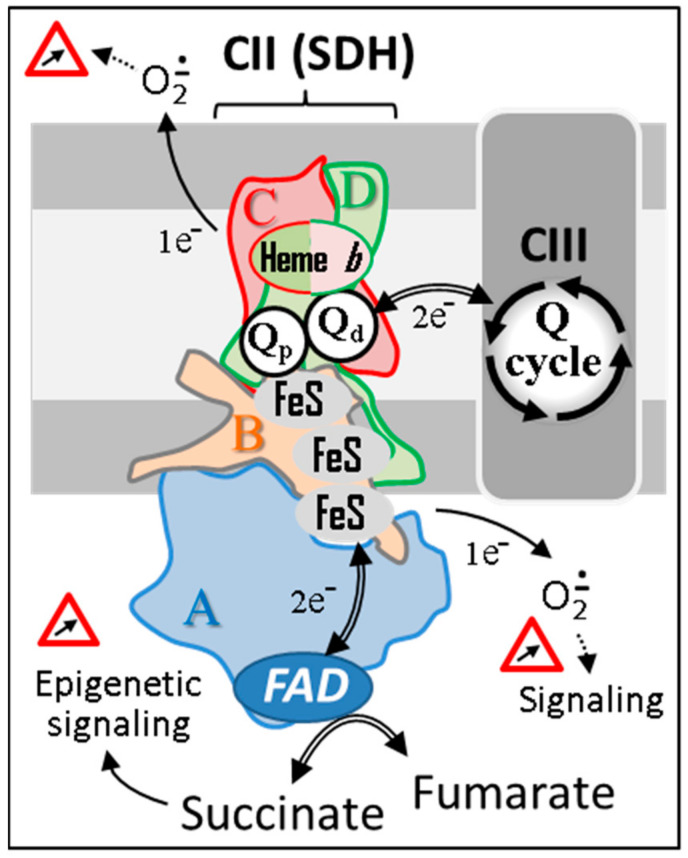
The succinate dehydrogenase. SDH’s four subunits (A–D) are conserved from microorganism to human. Animal SDH, as its plant counterpart, reduces a specific pool of ubiquinone that competes with other kinetically distinct quinone pools reduced by various mitochondrial dehydrogenases for the reduction of CIII-associated quinones. Depending on the reduction status of the redox centers of the enzyme, variable amounts of superoxides will be generated and used as physiological regulators of cell signaling pathways. However, in excess, superoxides can have multiple deleterious effects. Abbreviations/symbols: CII, CIII, respiratory chain complexes II, and III; FeS, iron-sulfur cluster; FAD, flavin adenine dinucleotide; Q, ubiquinone with variable length isoprenoid side chain according to species (CoQ_10_ in human).

In most microorganisms and animals, the enzyme comprises four subunits (Figure 1; SDHA to D or SDH1 to 4 according to organisms) all encoded by nuclear genes, typically conserved through evolution. These are organized as an operon in many prokaryotes [40]. Presumably, a further illustration of the archaebacterial origin of mitochondria [41,42,43], SDH3 and SDH4, are however still encoded by the mitochondrial genome in many but not all land plants [44]. Noticeably, the composition and function of the plant enzyme has been elusive and differs from the well-characterized enzymes in mammals and bacteria [45]. It appears to include up to four additional proteins [44,46], the function of which being yet unknown. SDH has also been reported to be associated with the thylakoid membranes of cyanobacteria [47], and with the chloroplasts of *Chlamydomonas* [48]. In higher plants, SDH identification in chloroplast preparations was attributed to the presence of contaminating mitochondria [49]. Noticeably, SDH is involved in several specific key roles in plants such as stomatal function and nitrogen assimilation [50].

The SDHA subunit, also known as the flavoprotein (Fp) subunit, harbors the catalytic/regulatory site(s) that binds organic substrates, succinate or fumarate, and various effectors, e.g., oxaloacetate, ATP, etc. [51]. SDHA is localized to the inner membrane where it faces, and protrudes into, the mitochondrial matrix space. Together with SDHB, it catalyzes the phenazine methosulfate-dichlorophenol indophenol (PMS-DCPIP) reductase activity without involving SDHC and D. These latter subunits are embedded in the inner membrane and bind a *b*-type cytochrome whose function(s) is not yet clearly determined [52]. SDHC and D subunits are often referred to as the anchoring subunits ensuring electron flow to ubiquinone. Noticeably, while other complexes of the respiratory chain may have gained additional subunits or contrariwise have lost some or all subunits (e.g., CI in *Saccharomyces cerevisiae*), SDH has a preserved composition across species with few exceptions. In addition to the four constitutive subunits, several supplemental assembly factors are required for the maturation of individual subunits and for the correct assembly and function of SDH. SDHAF1 (or LYRM8; SDH6 in yeast) is a chaperone that is involved in the maturation of SDHB by recruiting and inserting iron-sulfur clusters [53,54] while simultaneously affording protection from oxidative damages. It was suggested that a functional SDHAF1 necessitates SDHAF3 (SDH7 in yeast) [54]. SDHAF2 (SDH5 in yeast) appears to be required for the flavination of the SDHA subunit [55,56]. SDHAF4 (SDH8 in yeast) is known to bind to the flavinated SDHA subunit, affording protection from oxidants, while catalyzing the proper assembly of the catalytic dimer with SDHB [57]. A set of additional proteins ensures the proper functioning of SDH. These proteins encompass those involved in the assembly of iron-sulfur clusters, such as Frataxin [58]. The deficiency of Frataxin is known to cause Friedreich’s ataxia. This ataxia is hallmarked by a generalized deficiency of mitochondrial iron-sulfur cluster containing proteins, including SDH [59,60].

### 2.2. SDH Activity Assessment

**Figure 2:**
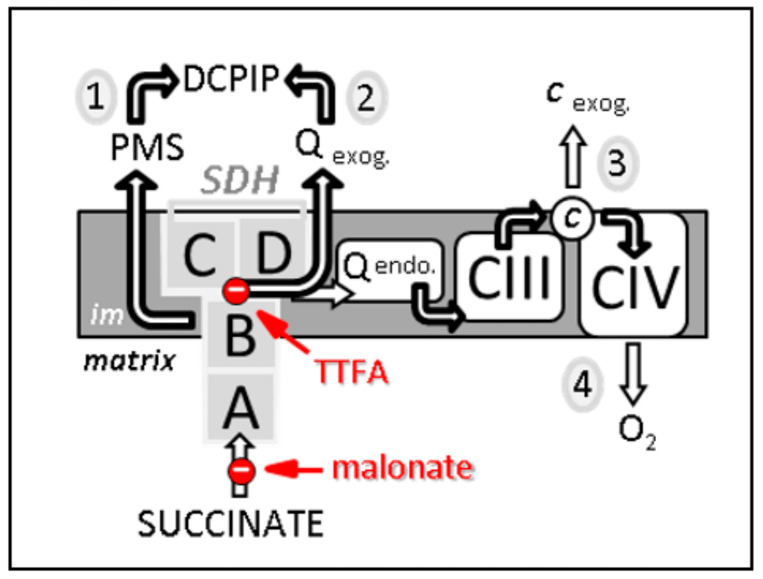
Four different methods, noticed 1 to 4, to estimate SDH-related activities. First, the measurement from succinate to DCPIP through PMS determines the electron transfer activity through SDHA and B subunits (noted 1 on the scheme). This activity is malonate-sensitive but TTFA-insensitive. Second, the reduction of DCPIP in the presence of the added short-chain homologue of ubiquinone, e.g., decylubiquinone, measures the activity of SDH through its four subunits A–D. This activity (2) is both malonate- and TTFA-sensitive. Third, the reduction rate of added cytochrome c by succinate (known as succinate cytochrome c reductase activity) measures the coupled activity of CII and CIII (3). This combined activity is often considered as a measure of SDH activity, which represents the rate limiting catalytic step (slowest step), making it rate limiting. This activity is sensitive to malonate, TTFA, and inhibitors of CIII, e.g., antimycin. Fourth, succinate-dependent oxygen consumption (4) measured either on isolated mitochondria, permeabilized muscle fibers, or various permeabilized cell types, can also be taken as reflecting SDH activity as long as the used conditions (e.g., mitochondria intactness, and non-limiting ADP availability) make SDH the limiting factor for oxygen consumption. Abbreviations/symbols: A–D, the four SDH subunits; *c*, cytochrome *c*; CIII, CIV, complexes III and IV of the respiratory chain; DCPIP, dichlorophenol indophenol; endo., endogenous; exog., exogenous (externally added); im, inner membrane of the mitochondria; PMS, phenazine methosulfate; Q endo., endogenous quinone, i.e., ubiquinone; Q exog., externally added quinones, e.g., decylubiquinone; TTFA, thenoyltrifluoroacetone.

An estimation of SDH activity can be achieved in different ways depending on the type of biological material available and on the nature of the question being asked (Figure 2). An SDH activity measurement can be carried out independently from any other element of the respiratory chain. This is generally carried out by estimating the reduction rate of an exogenous quinone, used as an electron acceptor, in the presence of the substrate of the enzyme, succinate.

This assay assumes that the enzyme is made freely accessible to the substrate and the acceptor. Therefore, it cannot be directly performed on intact cells or tissues. For that, plasma and outer mitochondrial membranes can be made permeable by using mild detergents such as digitonin or saponin that solubilize the cholesterol of the membranes. This step is coupled with the destabilization of the inner mitochondrial membrane by one or two freeze-thaw cycles [61]. DCPIP is used in an SDH activity assessment in order to keep the added quinones oxidized. DCPIP reduction is easily followed spectrophotometrically [62]. The use of a specific inhibitor such as malonate will allow one to verify the specificity of the measurement, that is, succinate oxidation. It is noteworthy that this assay should not be used to measure the sensitivity of the enzyme to inhibitors binding to the Q-binding domain of the enzyme such as SDHI (SDH inhibitor) fungicides, since they compete with added exogenous quinones to the Q-binding domain. As an alternative to avoid adding external quinones, it is possible to measure the combined CII + III activity. Indeed, in the presence of added cytochrome *c*, the terminal acceptor of CIII, SDH activity is estimated as succinate-cytochrome *c* reductase activity (SCCR) (Figure 2). SCCR activity is quantified spectrophotometrically by following the reduction of cytochrome *c*. This test is specific but requires verification that the activity of CIII is much higher than that of SDH; otherwise, the latter will be underestimated. Under these conditions, it becomes more realistic to measure the sensitivity of SDH to its inhibitors interacting with the quinone binding site, such as SDHI. This inhibition is now modulated by an inhibitor affinity for SDH and the endogenous quinone content. Finally, SDH activity may be measured under conditions entailing a preserved mitochondrial structure. In this case, it is possible to measure oxygen consumption related to succinate oxidation, using isolated mitochondria or cells where the plasma membrane, rich in cholesterol, is permeabilized by a small amount of digitonin. In this assay, a large part of the respiratory chain is involved (CIII, CIV, and CV). It is worth noting that under these conditions it should be checked that none of the respiratory chain segments involved are limiting for succinate oxidation.

Whatever the method chosen, it is judicious to couple SDH activity with that of additional enzymes under strictly the same conditions. The best case scenario is to run sequentially these measurements during one given test [62]. It must indeed be kept in mind that in many conditions the number of mitochondria may evolve considerably (up to 10 times) [63]. In spite of this variation, the ratios between the activity of the enzymes of the respiratory chain that are sequentially measured will remain globally unchanged [64].

### 2.3. A Peculiar but Meaningful Regulation

In mitochondria, under aerobic conditions, SDH catalyzes the oxidation of succinate to fumarate in the context of the metabolons that are known to orchestrate the protein-rich mitochondrial matrix (up to 500 mg/mL, i.e., close to that found in a protein crystal [65]). In this dense medium, enzymes and cofactors are functionally clustered, but not necessarily constantly and uniformly throughout the matrix space [66,67,68,69]. Electrons derived from succinate oxidation to fumarate are passed through a series of redox centers to a closely located quinone pool. Contrarily to other RC complexes, electron transfer through SDH occurs without proton pumping in the intermembrane space.

SDH appears to play largely similar and essential roles in all living organisms, which echoes its evolutionary conserved composition across species. This remarkable conservation undoubtedly accounts for the shared sensitivity of the enzyme to a wide variety of inhibitors, regardless of the organisms from which it originates [70]. Markedly, SDH is among the respiratory chain enzymes whose activities have been shown to be surprisingly heat resistant [71]. Accordingly, cellular meltome studies identified SDH subunits among the cell heat-resistant proteins [72]. The clustering of heat-releasing mitochondria in cells allows for heat-requiring processes to take place [73]. With a melting point close to 60 °C, SDH subunits have therefore the intrinsic capacity to work stably in a hot mitochondrial background reaching 50 °C; a temperature experimentally verified in a growing series of cell lines [74]. Interestingly, the gap between the high temperature values locally measured in mitochondria and the theoretical predictions is gradually fulfilled by the growing evidence of unexpected intracellular thermal conductivity variations [75]. This might fit with the idea that transient temperature spikes occur in order to allow for a significant heat differential between mitochondria, or part of it, and the surrounding cytosol under specific conditions [76].

**Figure 3:**
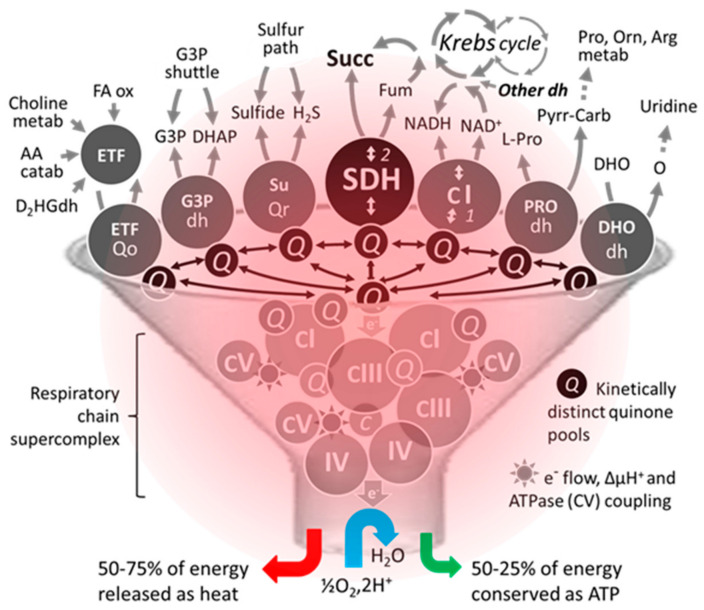
An unconventional schematic diagram featuring the succinate dehydrogenase among some of its competitors/coworkers for access to supercomplexes existing in the heating respiratory chain. The number of dehydrogenases capable of reducing part of mitochondrial ubiquinone varies between organisms. Moreover, their activity, distribution, and proportion can differ markedly depending on the organ, particularly in humans. Due to this competition, a small variation (<20%) can result in significant pathological consequences in humans [84]. Abbreviations/symbols: AA catab, amino acid catabolism; Arg, arginine; dh, dehydrogenase; DHO, dihydroorotate; DHAP, dihydroxyacetone phosphate; D2HGdh, D-2-hydroxyglutarate dehydrogenase; ETF, electron transfer flavoprotein; FA ox, β fatty acid oxidation; Fum, fumarase; G3P, glycerol-3-phosphate; H_2_S, hydrogen sulfide; metab, metabolism; Orn, ornithine; O, orotate; Pro, proline; Pyrr-Carb, pyrroline 5-carboxylate; Q, (ubi)quinone pool; Qo, quinone oxidase; Qr, quinone reductase; Succ, succinate; SuQr, sulfide quinone reductase; I-V, respiratory chain complexes I-V.

SDH, also known as RC complex II, is not part of the identified RC supercomplexes that involve varying combinations and ratios of complexes I, III, and IV [77]. Nevertheless, along with mitochondrial ATP-binding cassette protein 1 (mABC1), phosphate carrier, adenine nucleotide translocator, and ATP synthase, complex II or a portion of it has been reported to be part of a multiprotein complex (mitoK_ATP_) conferring ATP-sensitive K^+^ channel activity to mitochondria [78]. It should be noted that the composition of RC supercomplexes may vary with physiological conditions and can be even heterogeneous within one mitochondrion [79,80]. The identification of supercomplexes that kinetically insulate ubiquinone pools entails redox exchanges through spatially distant pools of ubiquinone [81]. The level of reduction of a given quinone pool is furthermore dependent on the kinetic properties of the dehydrogenase connected to the pool. Such kinetic compartmentation presumably holds true for all dehydrogenases, e.g., SDH (CII), glycerol 3 phosphate dehydrogenase, dihydroorotate dehydrogenase, NADH dehydrogenase (CI), dehydrogenases implicated in fatty acid oxidation, etc. (Figure 3). When active, all these dehydrogenases are susceptible to feed electrons to proton pumping RC supercomplexes for an ultimate reduction of oxygen to water by complex IV. Compartmentation also implies the coexistence of variably reduced ubiquinone sub pools within one mitochondrion. A locally highly reduced pool of ubiquinone may favor direct interaction with oxygen and the subsequent production of superoxides by an auto oxidation reaction. A high reduction of quinone may also render thermodynamically possible the reverse electron flow through redox centers, as long as an electron acceptor is quantitatively available. Typically, this type of reaction is favored by hypoxic conditions favoring quinone over reduction. Thus, driven by succinate, the reduction of ubiquinone by SDH can reverse the electron flow through CI resulting in the reduction of NAD^+^ [82]. On the other hand, in the presence of fumarate and reduced quinones, redox equilibria can convert the reaction of SDH (ΔG^0^ = 0 kJ/mol) into a fumarase reductase leading to the production of succinate [83].

Simultaneously to metabolic cooperation between substrates, various dehydrogenases compete somehow for transferring electrons to the terminal part of the RC as depicted in Figure 3 [84]. This also impacts the variable distribution of electrons from dehydrogenases to various terminal oxidases (obviously when these are present) as early reported in plant mitochondria [85]. Such kinetic compartmentation would be readily lost in the presence of an overpowered electron sink (e.g., high alternative oxidase, AOX, expression).

Unlike other RC complexes, SDH is activated under conditions where ubiquinone pools of the RC are rather reduced, e.g., under anaerobiosis. In addition, the activity of SDH is regulated by various cofactors. For instance, ATP binding to SDHA is known to upregulate SDH activity when mitochondria are under what is referred to as mitochondrial state 4 (high ATP/ADP ratio), which is marked by an increased proton motive force [86]. Conversely, oxaloacetate binding to SDHA downregulates or even fully inhibits SDH activity under state 3 (high ADP/ATP) [87,88]. OAA binds competitively to the succinate binding site [89] and regulates the rate of succinate oxidation [82,90].

Thus, SDH has the particularity to operate as much in the reduced state (state 4) as in the oxidized state (state 3). This impacts the status of the redox centers (1 Flavin, 3 FeS, 1 haem) that may be partially reduced under both state 3 and 4. The reduction level of such redox centers is known to control the production of superoxides by SDH. Thus, rather than the rate of electron flux through the enzyme, it is the enzyme reduction level that determines the magnitude of superoxide production [91]. Accordingly, a similar inhibition of electron flux brought about by malonate (binding on the substrate site on Fp subunit) and thenoyltrifluoroacetone (TTFA) (binding on the quinone-binding site of the enzyme) (Figure 2) results in opposite effects in terms of superoxide production due to divergent effects on the status of the redox centers. This potential uncoupling between rates of electron flow and levels of superoxide production makes it hazardous to predict and quantify the actual consequence of genetic mutations or chemical inhibitors.

An abundant literature is devoted to the role of superoxides in cell life, ranging from physiology to pathology [92]. Thus, through the production of superoxides, SDH may intervene in numerous key cellular processes. Moreover, in contrast to many RC-linked mitochondrial processes that tend to slow down under reduced conditions, which promote superoxide production, succinate dehydrogenase maintains a sustained activity and represents a perennial source of superoxides. However, this involvement in superoxide production must be closely regulated since superoxides are involved in many vital cellular processes including cell differentiation, cell proliferation, and cell death. Dealing with the peculiarities of superoxide production by SDH, different studies have been focused on the existence of micro domains involved in the production of superoxides at an enzyme level [93]. Accordingly, it was shown that a reverse electron flow through CI is not at the origin of superoxide production linked to succinate metabolism [94].

Like many enzymes of the intermediary metabolism, succinate dehydrogenase activity varies according to organisms and organs, and to their variable and fluctuating tissue- and cell-type specific metabolic demands [64]. Indeed, compared to the activities of the other complexes of the respiratory chain which appear to be mostly conserved between tissues, complex II activity varies in significant proportions among tissues [61]. For instance, SDH activity in the liver or kidney is greater than that of complex III, which allows SDH to remain in a reduced state, even when complex III is activated (phosphorylation state 3). Activities of other components of the RC may also vary among tissues (e.g., the glycerol 3-phosphate dehydrogenase, whose presence is not ubiquitous [64]), but their activities do not exceed the capacity of the RC to cope with their reducing activity. These enzymes remain presumably oxidized when the RC is freely working. The rate of quinone diffusion in the mitochondrial membrane between various dehydrogenases and RC complexes should also be taken into account. This rate may differ between tissues according to mitochondrial membrane composition and organization, being moreover affected by the long-ignored existence of super complexes in the respiratory chain. This discussion takes place because the reduction state of the redox centers of all these enzymes, up to eleven sites listed in mitochondria including SDH and G3Pdh, determines their propensity to generate superoxides, which are so important in cellular physiology [95].

## 3. Mitochondrial and Cellular Entangling

SDH links the activity of the Krebs cycle to that of the RC. The electron flow through SDH is not associated with a proton motive activity. Noteworthy, SDH does not participate in the RC supercomplexes. Moreover, it can initiate various reverse transfers of electrons under reduced conditions favoring the formation of superoxides. Indeed, the link between the phosphorylation process through the RC and the Krebs cycle appears to be particularly flexible. In contrast to most RC-linked enzymes, SDH activity is enhanced by a reduced environment favoring reverse electron transfer through complex I and the conversion of OAA, a major physiological inhibitor of SDH [89], to malate [82,96]. SDH activity is also enhanced by the binding of ATP to the enzyme [97], while in most segments of the respiratory chain, ATP promotes the reduction the electron transfer activity by preventing the dissipation of the proton gradient across the ATP synthase.

**Figure 4:**
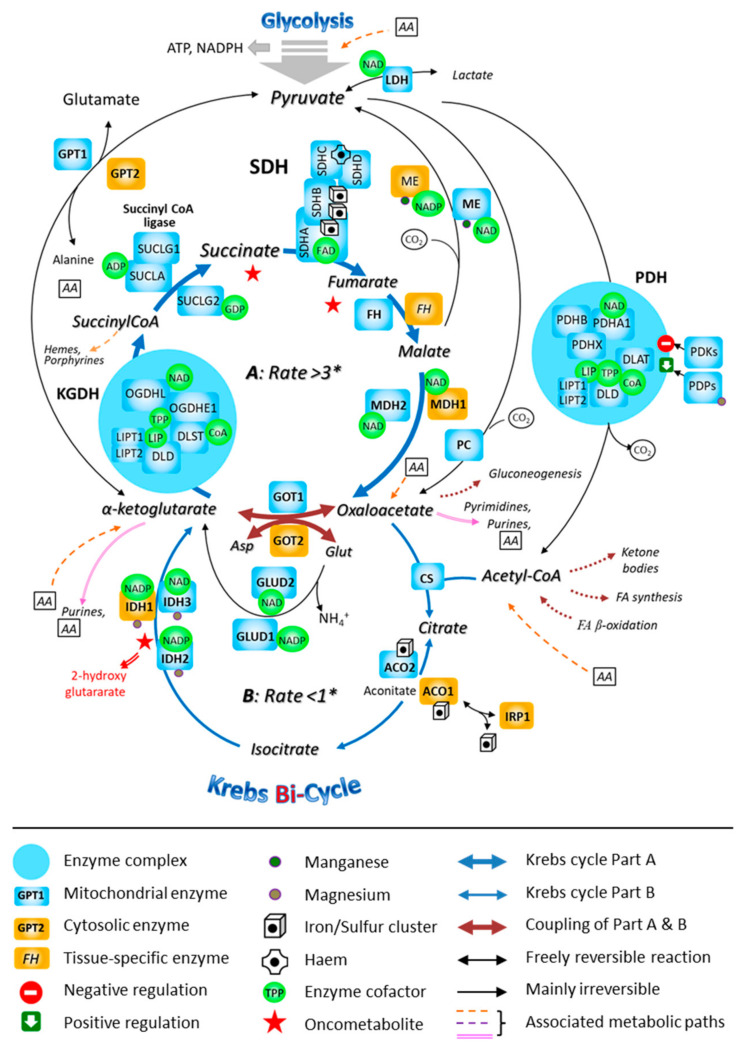
The involvement of the mitochondrial SDH in the fast segment of the Krebs bi-cycle. The Krebs cycle is represented as consisting of two segments, A and B, that are characterized by different measured flow velocities. * Respective fluxes through the segments A and B of the bi-cycle in nmol/min/mg prot. Abbreviations: ACO, aconitase; ADP, adenosine-diphosphate; FA, fatty acid; CoA, coenzyme A; CS, citrate-synthase; DLAT, dihydrolipoamide-S-acetyl-transferase (E2); DLD, dihydrolipoamide-dehydrogenase (E3); DLST, dihydrolipoamide-S-succinyl-transferase (E2); FH, fumarate-hydratase; GDP, guanosine-diphosphate; GPT, glutamate-pyruvate-transaminase; GOT, glutamate-oxaloacetate-transaminase; GLUD, glutamate-dehydrogenase; IDH, isocitrate-dehydrogenase; IRP, iron-responsive protein; LDH, lactate-dehydrogenase; LIP, lipoic acid; LIPT1, 2: lipoyltransferase; MDH, malate-dehydrogenase; ME, malic enzyme; NAD, nicotinamide-adenine-dinucleotide; NADP, nicotinamide-adenine-dinucleotide-phosphate; OGDH, oxoglutarate-dehydrogenase (α-ketoglutarate-dehydrogenase; OGDHE1: E1; OGDHL, E1-like); PC, pyruvate-carboxylase; PDH, pyruvate-dehydrogenase (PDHA1 and PDHB, α and β subunits of E1); PDHX, pyruvate-dehydrogenase compound X; PDKs, pyruvate-dehydrogenase-kinases; PDP, pyruvate-dehydrogenase-phosphatase; TPP, thiamine-pyrophosphate; SDH, succinate-dehydrogenase; SUCL, succinyl CoA-ligase.

Although the Krebs cycle was initially described as forming a single entity, it appears that it is actually made up of at least two segments (Figure 4), that are distinguished by specific flow kinetics [98]. The segment of the Krebs cycle in which SDH is engaged represents a path through which glutamate (possibly arising from glutamine) can be substituted for acetyl-CoA (possibly arising from pyruvate) as a carbon source for the Krebs cycle. The multiplicity of metabolic pathways and the spatial organization of matrix proteins into metabolons have been studied and described in the mitochondria from only some tissues of few species. Indeed, this intra-mitochondrial complexity further foreshadows the multiple possibilities of interaction within the changing cellular metabolic context in which the mitochondria work. Hence, reducing the functioning of the mitochondria to a simple scheme that can be generalized to all situations, all organisms, and their organs is unreasonable. Some functional elements will however be found in all mitochondria, but their layouts and activity levels vary in very significant proportions [64].

A rapid survey of the scientific literature of the last twenty years shows that the succinate level and the activity of the enzymes and proteins producing it, using, or targeted by this metabolite, as well as the succinate-dependent redox signals, have to be very tightly controlled in most organisms [23,99,100]. This highlights the peculiarity and strategic role both the organic acid itself and associated enzymes play in cellular physiology.

## 4. Genetics and Epigenetics: SDH and Succinate

The genes encoding SDH subunits have now been sequenced in many species, ranging from microorganisms to higher mammals. A comparative sequence analysis indicated a spectacular conservation of the genes encoding the four core subunits of the enzyme with slight noted variations [70]. Paralogs of SDH genes (*Sdh3* and *Sdh4*) have been reported in *Saccharomyces cerevisiae* [101], while the expression of a dispensable *Sdh3* paralog has been shown to account for resistance to the SDH inhibitor in another fungus, *Zymoseptoria tritici* [102]. Various mechanisms, i.e., alternative splicing [103], promoter usage [104], or post-transcriptional modifications [105], contribute significantly to the large variation in the expression patterns of SDH subunits. Noticeably, alternative splicing of *SDHC* has been described in humans and is reported to play a role in rare human pathologies [103].

The metabolism sustains cellular life. In turn, cells must sense the nutritional status. This is done through multiple signaling networks. Accordingly, numerous chromatin-modifying enzymes respond to metabolites and metabolic enzyme cofactors. For instance, it has been shown that Histone Acetyltransferase uses Acetyl-CoA as a cofactor, while PARPs (Peroxisome proliferator-activated receptors) and Sirtuins activity are dependent on Nicotinamide Adenine Dinucleotide (NAD), Lysine demethylase 1 on Flavin Adenine Dinucleotide (FAD), DNA, and Histone methyltransferases on S-adenosyl-methionine (SAM) [106]. Moreover, the activities of several dioxygenases catalyzing the α-ketoglutarate-dependent hydroxylation of proteins (e.g., HIF 1 α), as well as the activities of the TET-family DNA demethylases and of Jumonji C-family Histone demethylases are sensitive to the α-ketoglutarate/succinate balance. In humans, the specific role of succinate in these crucial reactions was brought to light when disease-causing pathogenic variants in genes encoding SDH subunits were uncovered [15,107,108]. Mitochondrial metabolites and cofactors have until now been seen primarily as sources and regulators of cellular energy through ATP synthesis. We now know that they play infinitely more complex roles that are still largely to be dissected [5]. These long-ignored mechanisms control through epigenome remodeling, and in a very coordinated manner, mitochondrial activities, cell proliferation, and death, as well as cell differentiation in all living organisms [109].

Most chromatin-modifying enzymes use important intermediates of the mitochondrial and cytosolic metabolism as cofactors. Depending on dietary intake, the level and distribution of metabolites will vary and will therefore affect the expression of a large number of genes by modulating the activity of epigenetic pathways and associated chromatin-modifying enzymes. Considering that the methylation of DNA and each post-translational modification (PTM) of proteins can be affected by many metabolic pathways, the epigenome might act as a sensor of the whole metabolic network.

As a result of a decreased activity of any of the Krebs cycle enzymes, the overall activity of the cycle may decrease, as long as the affected enzyme controls the rate of the segment where it acts. Simultaneously, a subset of the cycle intermediates may escape the mitochondrial matrix and accumulate in the cytosol resulting in an imbalance of organic acids within the mitochondria and more generally in the cell. In case of an SDH blockade, it was established as early as 2005 that succinate accumulated in cells to an extent that disrupted the equilibrium with α-ketoglutarate, which altered the activity of the prolyl-hydroxylase that controls the hypoxia-sensitive pathway [110]. This disruption is now recognized as triggering tumorigenesis and cancer [111,112].

## 5. An Unexpected Spectrum of Human Diseases

Heterozygous germline mutations in SDH complex (*SDHx*) genes (*SDHA, SDHB, SDHC, SDHD*, and *SDHAF2*), which act as tumor suppressor genes, predispose to pheochromocytomas and paragangliomas (PPGLs) and rarely to gastrointestinal stromal tumors (GIST), renal cell carcinoma, and pituitary adenomas. *SDHx* genes’ associated germline mutations (substitution or deletion) have an autosomal-dominant inheritance, which implies the occurrence of a secondary somatic genetic event at specific gene locus (loss of the wild type allele or loss of heterozygosity, another genetic variant in trans allele, promoter hypermethylation, or epimutation) to trigger tumorigenesis. The first mutations in an *SDHx* gene (*SDHD*) have been reported in patients with PPGL in 2000 [113]. Today, it is believed that 40% of PPGLs are inherited and that about half of these carry a germline mutation in genes encoding tricarboxylic acid enzymes such as succinate dehydrogenase (SDHA, SDHB, SDHC, SDHD, SDHAF2), fumarate hydratase (FH), malate dehydrogenase (MDH2), and the dihydrolipoyl lysine-residue succinyl transferase component of 2-oxoglutarate dehydrogenase complex (DLST), as well as components of the malate-aspartate shuttle such as the oxoglutarate/malate carrier (SLC25A11) or the mitochondrial aspartate aminotransferase (GOT2), which all lead to cancer via different mechanisms including epigenetic modifications [114].

Paragangliomas are rare neuroendocrine tumors that can develop in parasympathetic and sympathetic paraganglia, from the skull base to the pelvic region, while pheochromocytomas arise in adrenal medulla. Catecholamine-producing tumors (pheochromocytoma and functional paraganglioma) are severe disorders, sometimes revealed by life-threatening emergencies or by hypertension and/or cardiovascular morbidities. Non-secreting paragangliomas are most frequently located in the head and neck and are revealed by a cervical mass or a hearing loss. International recommendations stated that affected patients should be referred to multidisciplinary expert centers for imaging, treatment, and management. Surgical resection or therapeutic radiations are usually discussed as first options [99]. PPGLs become metastatic in around 15% of the cases [115].

A study of patients with *SDHx*-related PPGL has revealed interesting, yet mysterious, phenotype-genotype correlations. Patients with *SDHD* pathogenic variants develop predominantly multiple head and neck tumors, whereas patients carrying *SDHB* pathogenic variants develop usually extra-adrenal, retroperitoneal, or pelvic paragangliomas. The identification of a germline mutation in the *SDHB* gene is a risk factor for malignancy. Nevertheless, whatever the mutated *SDHx* gene, mutation-carriers can present on all of the disease spectrums. An international consensus on initial screening and follow-up of asymptomatic *SDHx* mutation carriers was published in 2021 [112,116].

Another *SDHx*-related neoplasia is the gastrointestinal stromal tumors commonly referred to as succinate dehydrogenase (SDH)-deficient GISTs. Around 50% of SDH-deficient GISTs are caused by hypermethylation of the *SDHC* promoter (epimutation); 30% by a germline mutation in *SDHA*; and 20% to 30% in *SDHB*, *SDHC*, or *SDHD*. SDH-deficient GISTs are usually diagnosed in children and young adults. They appear to be specifically located in the stomach. Frequently indolent, they can be multifocal and metastatic. SDH-deficient renal cell carcinoma is rare, accounting for less than 1% of renal carcinomas. In most of the cases, they are caused by an *SDHB* gene mutation and diagnosed in a young adult [117]. SDH-deficient pituitary adenomas are benign tumors, treated by surgery and/or dopamine agonists, which can produce prolactin and/or growth hormone or be non-functional. Only in a very few cases, has the causality between *SDHx* variants and pituitary adenoma been clearly established [118]. SDHB immunohistochemistry is a well-validated tool for detecting SDH-related neoplasia after surgery. Whatever the mutated SDH subunit, a loss of protein B expression is observed in the tumor tissue. Furthermore, SDHA immunohistochemistry detects SDHA-related tumors specifically [119]. Tumor metabolite profiling also shows a high diagnostic performance and constitutes an efficient alternative method [120]. Recently, an in vivo approach has been developed to confirm the *SDHx*-mutated status of a tumor by proton magnetic resonance spectroscopy, which is able to detect a succinate peak testifying to the succinate accumulation in the tumor resulting from succinate dehydrogenase inactivation [121].

The last forms of SDH-deficient diseases are mitochondrial complex II deficiencies causing early-onset progressive neurodegenerative autosomal recessive disorders due to homozygous or heterozygous composite *SDHx* pathogenic variants. Most clinically affected individuals reported in the literature harbor pathogenic variants within the *SDHA* gene and present with a Leigh syndrome, epileptic encephalopathy, and cardiomyopathy [122]. Less common, pathogenic variants involving *SDHB*, *SDHD*, and *SDHAF1* genes have also been reported [25,123]. The first recessive *SDHA* gene mutation was reported in two siblings diagnosed in early childhood for a typical Leigh syndrome, hallmarked by the hypodensity of the white matter revealed by a computer tomography scan (CT scan). They both presented a rather similar disease course, developed normally up to 10 months, when they rapidly presented marked rigidity, bilateral pyramidal tract signs, cortical blindness, and difficulties in swallowing fluids. They died in their second year of life. The mutation caused a severe, yet partial, deficiency of SDH activity, resulting in a tissue-specific involvement [3]. Bi-allelic genetic variants were reported in *SDHB* in a dozen patients developing infantile leukoencephalopathy [124]. Compound heterozygous mutations in the *SDHD* gene were first reported in a girl with encephalomyopathy and biochemical evidence of isolated mitochondrial complex II deficiency who died at 10 years, and homozygous *SDHD* mutation in an infant with fatal cardiomyopathy and mitochondrial complex II deficiency [125,126].

## 6. A Worrisome Environmental Context

Succinic acid is a natural constituent of basically all organic tissues, varying according to time and conditions. Its distribution is not only limited to organisms but also extends to their biological products, e.g., honey [127]. Succinate plays fundamental roles in many key molecular, cellular, and physiological processes. Modulating the supply of succinate represents, therefore, a means by which the physiology of organisms may be affected. Thus, succinate made available to gut microorganisms can also affect the physiology of their host [128,129]. Succinate is quite stable. Baltic amber, resulting mainly from the fossilization of the conifer resin, contains up to 8% of it in its surface, hence the name amber acid sometimes given to succinate [127]. It is also worth mentioning that a significant number of virtues especially in term of human health have been attributed to succinate, which enabled a sustained trade for many years. Currently, succinate is commercially produced and approved by the US Food and Drugs Administration.

### 6.1. Natural SDH Inhibitors

**Figure 5:**
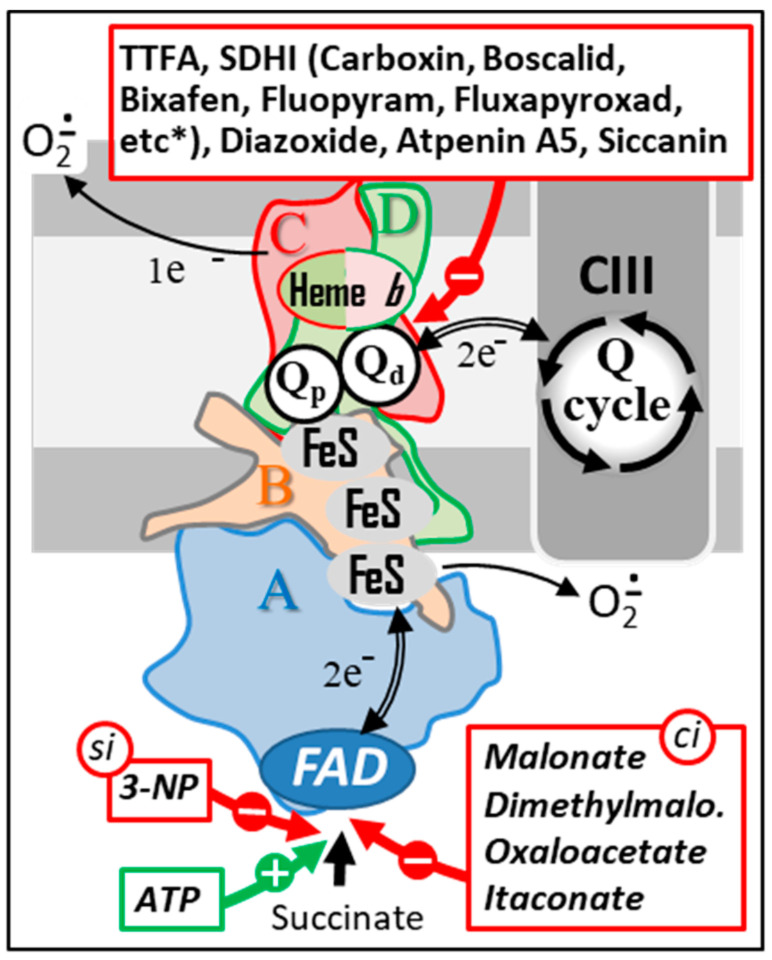
Natural and chemical SDH inhibitors. Sites of action of a series of the best-known SDH inhibitors are indicated by red arrows. A subset of these inhibitors (bottom) acts either as competitive inhibitors (ci) or as a suicide inhibitor (si) by binding to the SDHA subunit. ATP, which is also capable of binding to this same subunit, acts as an activator of SDH activity (green arrow). Another group of inhibitors act by interacting with the endogenous quinone binding site (top). This type of inhibitors includes a large number of molecules used as fungicides in agriculture (a more complete list of SDHI inhibitors can be found at http://endsdhi.com/wp-content/uploads/2022/04/SDHI-structure-15-Avril-22.pdf, accessed on 22 July 2022). Abbreviations/symbols: ATP, Adenosine triphosphate; CII, CIII, respiratory chain complex II and III; FeS, iron-sulfur cluster; FAD, SDH A-bound flavin adenine dinucleotide; 3-NP, 3 nitropropionic acid; Q, ubiquinone with variable length isoprenoid side chain according to species (CoQ10 in human); Qp, Qd, proximal and distal Q binding sites, respectively.

Considering the crucial role of succinate metabolism, it is not surprising that many organisms, including humans, attempt to get rid of predators and pests by targeting their ability to metabolize succinate. Among the various mitotoxic and oxytoxic compounds targeting diverse mitochondrial functions and cell oxygen metabolism, many plants produce a subgroup of compounds susceptible to target SDH (Figure 5). Thus, malonate accumulates in several plant families, frequently in leguminous plants. In chickpea, malonate is the predominant acid in roots and nodules, but not in leaves. This has been taken as an indication of a defensive role in these plant parts [130]. On the other hand, several bacteria, fungi, mammals, and plants possess the ability to metabolize malonate [131,132]. Malonate might also have beneficial effects. Intracoronary-injected malonate, as well as dimethyl malonate which can be found in several plants (e.g., *Ananas comosis, Myrtus communis Astragalus*), affords cardio protection against ischemia-reperfusion injury. However, due to its toxic side effects, malonate systemic administration in animals is precluded [133,134]. Atpenin A5, another substance, is known as an antifungal antibiotic that inhibits SDH activity. Atpenin A5 is also quite active against nematode and human SDH (respectively, IC_50_ = 12 and 3.7 nM). It was initially extracted from *Penicillium* sp. Similar to malonate or dimethyl malonate, Atpenin A5 is cardio protective. The cardio protection afforded by these SDH inhibitors has been ascribed to a potential activation of mitochondrial K^+^ channels whose relationship with SHD remains obscure [135,136]. Moreover, the modulation of SDH activity by these effectors has an unpredictable cardioprotective effect [137]. Noticeably, these molecules do not similarly bind to SDH. While malonate is well known as a competitive inhibitor of the enzyme at the substrate-binding site, Atpenin A5 is a highly specific ubiquinone-binding site. Binding to these two opposite sites on SDH has been reported to have opposite effects in terms of superoxide production. Since malonate and Atpenin A5 act on the two opposite inhibitor sites of SDH (Figure 5), it appears difficult to envision the modulation of superoxide production (by SDH) as instrumental in cardio protection, since these two inhibitors have a dissimilar outcome.

Several other SDH inhibitors have been identified, mostly in plants or microorganisms. For example, 3-nitropropionate, also known as Bovinocidin, or Hiptagenic acid, is a glycoside found in numerous fungi. It is also found in the upper part of many plants where it is readily liberated in the gut of herbivores, allowing it to exert its toxic effect [138,139]. Nitro propionate is noticeably present in the poisonous plant *Indigofera tinctoria* used to prepare indigo dyeing [140]. It is a suicide inhibitor of SDH which displays antimycobacterial properties and is used for skincare. This toxin can also originate from food contaminated by *Aspergillus* sp. Nitro propionate has been used to generate models of Huntington’s disease, a neurodegenerative disorder characterized clinically by subtle problems with mood or mental abilities followed by a general lack of coordination and an unsteady gait [141]. In rats, Nitro propionate resulted in behavioral anomalies, with somnolence and general depressed activity, ultimately leading to coma and mimicking, to a certain extent, Huntington’s disease [142]. Nitro propionate was also used in mice, where it was shown to cause ataxia [143], in *Drosophila* [144], and in baboons [145]. Siccanin is another SDH inhibitor, initially isolated from a pathogenic fungus, that has been recently proposed as a new chemotherapeutic agent [146]. Promysalin, a secondary metabolite targeting SDH, produced by the bacteria *Pseudomonas putida* has been proposed to inhibit the growth of another *Pseudomonas,* namely the Gram-negative pathogen *Pseudomonas aeruginosa* [147,148]. SDH inhibitors are also found as secondary metabolites in various strains of *Penicillium roqueforti* used in the famous French Roquefort cheese.

### 6.2. Chemical Inhibitors: Poisons and Medicines

Beyond these naturally occurring SDH-inhibitors, always locally confined, the agrochemical industry produces and recommends the widespread use of SDHIs to counteract fungi or nematodes proliferation on seeds, plants and their product, fruits, and vegetables. SDHIs just in a few decades have been massively disseminated in nature, from now on impregnating living organisms and the entire biosphere. The dissemination of unspecific pesticides such as SDHIs, among various pesticides, already has devastating consequences, illustrated by the major collapse of insect and bird populations, to only mention the most visible concerned organisms [149,150]. It is also important to note that it is not really feasible to quantify the impact of SDHIs on the soil micro-organisms in terms of their populations and respective equilibria. Concerning human health, it has become a nightmare to estimate the impact of any substance, including SDHI molecules, taking into account the number of molecules utilized, their frequent changes, and the variable exposition of the population.

Due to their generalized use, SDHIs are now omnipresent in the various compartments of the biosphere (earth, water, air), including protected areas [149]. The generally low concentration of these inhibitors may be functionally counterbalanced by the simultaneous presence of several - dozens, or more, of toxic molecules [151], in particular of compounds affecting either the use of oxygen (mitotoxics targeting mitochondria) or oxytoxics, modifying the generation of the detoxification of reactive oxygen derivatives. The role of this pesticide mixture is regularly pointed out in the biodiversity loss. It may also have a role in the evolution of various human diseases where mitochondrial, especially SDH, activity is impaired [152]. Indeed, while human diseases originating from SDH gene mutations/losses are now fully recognized, the mechanism of disease progression is far from being understood. As in most mitochondrial diseases, variable expression, or variable time of onset, even within a given family, has been observed. Thus, other than individual genetic factors, this leaves room for disease modulation by additional external factors.

In that regard, to only speak of the diseases where SDH is affected, altitude has been identified through population studies as a phenotypic modifier in hereditary type 1 paragangliomas, resulting from *SDHx* gene mutations, with evidence pointing to an oxygen-sensing defect [153]. Dysregulation of HIF1α has been further recognized as instrumental for this oxygen-sensitive process. Oxygen has also been recognized as a phenotype-modifier in a mitochondrial disease such as Friedreich ataxia, where SDH is involved and the signaling of cell antioxidant defenses is being weakened [154]. More generally, environmental-induced oxidative stresses are known as key actors in the course of mitochondria-associated neurodegenerative disorders and aging [155].

The widespread use of SDHI is known to result in the rapid appearance, in a few years, of numerous mutated and resistant fungal species [156,157]. This makes the long-term use of each SDHI problematic and results in a faster turnover of SDHIs of the last generation that were shown to interfere with other ubiquinone-binding proteins, such as those involved in complex III [152]. On the other hand, the widespread use of SDHIs increases the risk of the appearance of dangerous drug-resistant microorganisms, including fungi [156,158,159]. These, with potential growth advantages, could in the future become multi-drug resistant and have uncontrollable broadening targets, from plants to other living organisms.

## 7. Concluding Remarks

The conclusion that emerges from this review covering a wide field of scientific knowledge is that we face more questions than answers, despite the colossal work conducted by so many scientists on SDH and its substrate succinate for nearly a century. This stands true whatever the angle from which the subject is approached, whether starting from the enzyme or its substrate.

To start from the most fundamental aspect: the transfer of electrons through the SDH still retains a part of the mystery. For example, the function of the haem component of SDH is still hypothetical. Does it act as a source or a trap vis-à-vis superoxides, or both? Moreover, several redox centers have the capacity to generate superoxides, but which one of them is involved in this production is not known in many conditions. Furthermore, the production of superoxides depends on the redox state of these centers, but what is the extent of the relationship between the speed of the flow of electrons through the enzyme and the reduction state? In other words, the relationship between the activity level of the complex and the production of superoxides remains to be established.

Another unsettled question concerns the interactions of SDH with other complexes and supercomplexes of the RC, or other constituents of the inner mitochondrial membrane and the matrix. It should be, however, emphasized that such interactions may vary over time depending on the conditions, the organisms, the organs, or even depending on the location of the enzyme in the heterogeneous mitochondrial space. The oversimplification of these aspects, often implemented for honorable pedagogical reasons, ends in unrealistic structural and functional models that do not correspond in any way to the complex and variable reality.

Concerning the dichotomy of clinical presentations associated with mutations in the various SDH encoding genes, primarily discussed on the basis of the SDH subunit genes found mutated [160], it should be firstly noted that additional clinical presentations have also been reported, e.g., isolated cardiomyopathy [126,161]. Then, it is worth noting that similar contrasting clinical presentations have been reported associated with mutations in most of the enzymes of the Krebs cycle, including fumarase, a tetrameric enzyme encoded by a single gene [2,162].

Regarding the substrate of SDH, succinate, it has been known for a long time to be a key intermediate of the mitochondrial metabolism and consequently of the whole intermediary metabolism. Succinate also has a well-established role in the mitochondrial oxygen-dependent energy production in the form of heat or ATP. Once again, this role is variable according to the organisms, organs, and situations, resulting in a tangle that is difficult to reduce to oversimplified metabolic diagrams. The question is far from academic at a time when a computer-assisted in silico representation of metabolism based on such diagrams is proposed to shed light on the effect of inhibitors on the metabolism [163]. Thus, it has been “demonstrated” that a blockade of SDH (in this instance, by SDHI) in humans should not result in succinate accumulation, ignoring the large number of studies that have measured this accumulation resulting from inhibitors or genetic anomalies [106,110,123,164,165,166,167,168,169,170]. Such an accumulation is now used routinely to detect tumors linked to SDH defects [121].

We now also know that succinate is a key oncometabolite acting in both cell differentiation and proliferation, due particularly to its effect on several methylases involved in controlling the expression of the genome. In this way, the metabolic balances involving succinate play a crucial role during the development of living organisms. Through succinate-dependent prolyl hydroxylases, it also controls the response to factors important for aerobic organisms and their various responses to hypoxia. Again, the entanglement of functions involving succinate, even considering only a single organism, appears such that it cannot be summarized in oversimplified diagrams.

In such a context, one should understand, as Singer and colleagues did 40 years ago [171,172], the major risk for the entire biosphere, including man [173], of using this metabolic crossroad as a target for any large scale or long duration treatment.

## Data Availability

Not applicable.
